# Durations and Delays in Care Seeking, Diagnosis and Treatment Initiation in Uncomplicated Pulmonary Tuberculosis Patients in Mumbai, India

**DOI:** 10.1371/journal.pone.0152287

**Published:** 2016-03-28

**Authors:** Nerges Mistry, Sheela Rangan, Yatin Dholakia, Eunice Lobo, Shimoni Shah, Akshaya Patil

**Affiliations:** 1 The Foundation for Medical Research, 84A, R. G. Thadani Marg, Worli, Mumbai, India; 2 Maharashtra Association of Anthropological Sciences-Centre for Health Research and Development, Savitribai Phule University, Pune, Maharashtra, India; Universidad Nacional de la Plata, ARGENTINA

## Abstract

**Background:**

Timely diagnosis and treatment initiation are critical to reduce the chain of transmission of Tuberculosis (TB) in places like Mumbai, where almost 60% of the inhabitants reside in overcrowded slums. This study documents the pathway from the onset of symptoms suggestive of TB to initiation of TB treatment and examines factors responsible for delay among uncomplicated pulmonary TB patients in Mumbai.

**Methods:**

A population-based retrospective survey was conducted in the slums of 15 high TB burden administrative wards to identify 153 self-reported TB patients. Subsequently in-depth interviews of 76 consenting patients that fit the inclusion criteria were undertaken using an open-ended interview schedule. Mean total, first care seeking, diagnosis and treatment initiation duration and delays were computed for new and retreatment patients. Patients showing defined delays were divided into outliers and non-outliers for all three delays using the median values.

**Results:**

The mean duration for the total pathway was 65 days with 29% of patients being outliers. Importantly the mean duration of first care seeking was similar in new (24 days) and retreatment patients (25 days). Diagnostic duration contributed to 55% of the total pathway largely in new patients. Treatment initiation was noted to be the least among the three durations with mean duration in retreatment patients twice that of new patients. Significantly more female patients experienced diagnostic delay. Major shift of patients from the private to public sector and non-allopaths to allopaths was observed, particularly for treatment initiation.

**Conclusion:**

Achieving positive behavioural changes in providers (especially non-allopaths) and patients needs to be considered in TB control strategies. Specific attention is required in counselling of TB patients so that timely care seeking is effected at the time of relapse. Prioritizing improvement of environmental health in vulnerable locations and provision of point of care diagnostics would be singularly effective in curbing pathway delays.

## Introduction

Every year approximately 1.5 million people die of Tuberculosis (TB) globally, and most of these occur in the developing countries. [1] India is one of the 22 TB high burden countries in the world; approximately 2.2 million cases in 2014 out of the estimated annual global incident cases of 9.6 million were reported from India. [2] Mumbai, the financial capital of India, with a population of approximately 14 million, 50–60% of whom live in overcrowded slums with extremely poor hygiene, sanitation and ventilation, [3, 4] is a hotspot for multi and extensive drug resistant TB in the country. This has been documented by numerous studies in the last decade. [5, 6, 7, 8]

According to Madebo *et al*, delay in diagnosis and subsequent treatment initiation plays a major role in increasing the infectious nature of the disease.[9] In high burden countries like India, the duration for diagnosis and initiation of treatment is noted to be higher, and is attributed to both patient- and provider-related factors.[10,11] For effective and significant control of TB, early care seeking by patients, accurate diagnosis and prompt initiation of treatment is paramount.[12,13] This is especially important for vulnerable populations like slum dwellers, who form the low socio-economic and underserved sections of the community. [14, 15]

Evidence from more than 23 studies on delays in TB care in India have documented delays in first care seeking ranging from 6 to 268 days, delay in diagnosis from 4 to 268 days and delays in treatment initiation from 1 to 8 days.[16] Four studies on delays have been conducted in Mumbai in the last 5 years. [15, 17, 18, 19] Most of these studies were conducted at facilities under the Revised National TB Control Programme (RNTCP). Such studies may be prone to respondent bias, and furthermore miss out patients seeking care in the heterogeneous private sector consisting of formal and non-formal providers, where more than 60% of patients in India seek health care. These studies may therefore depict an incomplete picture of the actual scenario of delays. [16, 20, 21, 22] Our study, in contrast, was designed as a community-based survey for capturing information on pathways of self-reported pulmonary TB patients, thereby eliminating the inherent bias of facility-based studies.

Mumbai is among the first cities in the country to have public-private mix (PPM) interventions started under the RNTCP.[23] However despite these efforts in the past decade, the latest studies from India continue to document significant delays in TB diagnosis and treatment initiation [15,16,17,18,19]. Our study was conducted as a baseline situational analysis for a Public-Private Interface Agency (PPIA) intervention planned to be implemented in Mumbai. The objectives were to provide real-time data and fill-in gaps from past studies on time taken and factors responsible for delay in first care seeking, diagnosis and treatment initiation in a vulnerable urban setting.

## Methods

### Study design, setting and sampling

A population-based, two-stage, retrospective study was conducted between April and July 2014.

The sampling universe comprised of slums from 15 high burden administrative wards for TB and MDR-TB identified by the Municipal Corporation of Greater Mumbai (MCGM).

The first stage involved initial household (HH) survey to identify known TB cases using a multistage cluster approach [24] framed on the 2011 Census Enumerated Block (CEB) maps.

The HH survey was conducted using an electronic questionnaire displayed on a mobile/PDA developed in Census & Survey Processing System (CSPro) v5. It captured information provided by respondents regarding TB diagnosis, treatment and any available documentary evidence. The survey of 14,250 participating households, yielded 153 TB patients.

Trained public health researchers conducted the second stage, which comprised of in-depth interviews of these TB patients. The inclusion criteria consisted of consenting patients of all ages and sexes diagnosed and treated for pulmonary TB (PTB) in Mumbai or patients who had completed their TB treatment in the past six months.

### Data collection

**Fig 1:**
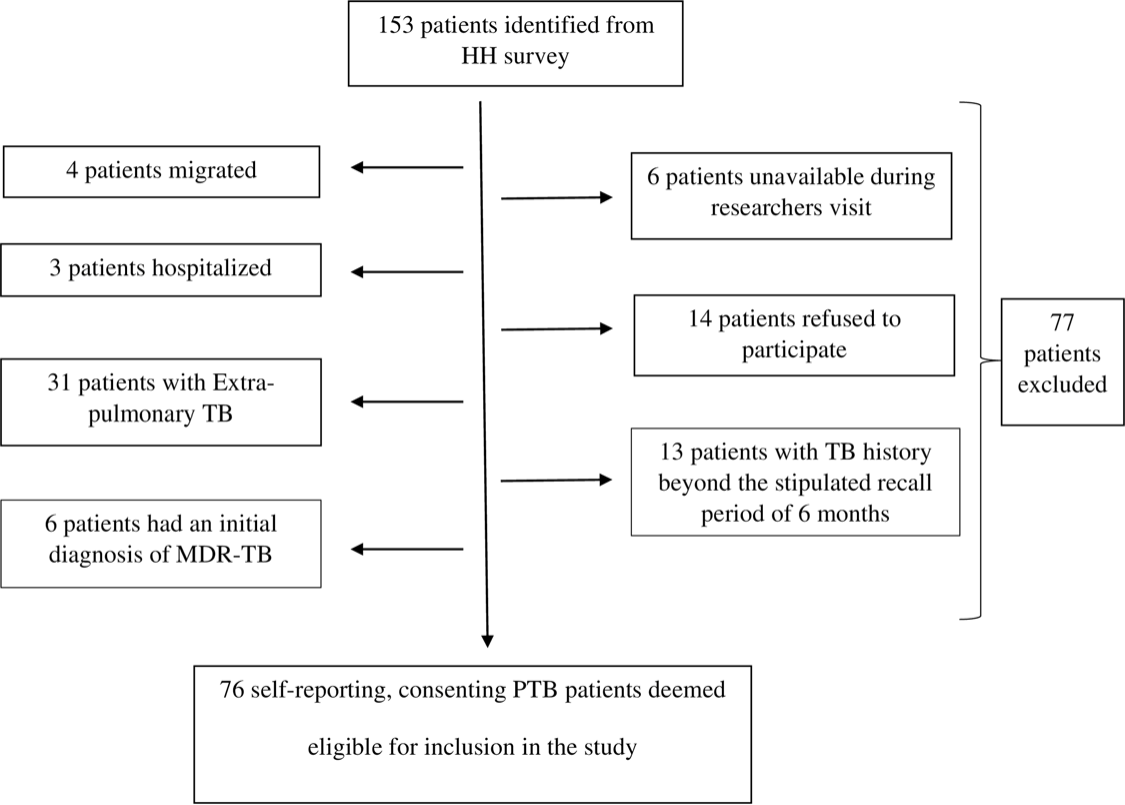
Patients selection for in-depth interviews. The figure lists the various reasons why identified TB patients were not included for the in-depth patient interview.

On obtaining details from the HH survey team, the researchers contacted the patients within 3–4 days, either via telephone or household visits for fixing appointments and/or conducting interviews. Of the 153 TB patients identified through the initial HH survey, 77 patients could not be interviewed (Fig 1). Patients unavailable due to reasons such as hospitalization or unavailable at the time of researchers’ visit were contacted more than once for the interview. A maximum of three attempts were made to contact and interview such patients. The final sample therefore included 76 self-reporting, consenting PTB patients deemed eligible for inclusion in the study.

After obtaining informed consent, the respondents were interviewed by a pair of researchers. For minimization and estimation of recall bias, patients’ documents with dates were checked prior to the interview to triage findings, interviews were conducted in the presence of family members (parents/spouse/children), and calendars with relevant dates including holidays, religious festivals etc. were used to pinpoint events. All in-depth interviews were conducted at the patients’ residence or a location of their convenience at a time and date preferred by them, and in the local language. The approximate duration of interviews was 60 to 90 minutes. Privacy was ensured, wherever possible, by conducting the interviews in a location where people other than family members could not overhear or contribute to the interview.

Participants were compensated for their time with a kit containing grocery items and personal hygiene products.

### Study tools

In-depth interviews were conducted using a pre-tested open-ended interview schedule. The schedule was first developed in English, translated into local languages (Hindi and Marathi) and then back translated into English to check for consistency. A comprehensive quantitative data sheet was also developed for entering all the quantitative data collected from the in-depth interviews. Audio recording of interviews were done after obtaining the patients’ permission.

The key quantitative themes covered in these interviews were: the time taken from onset of symptoms suggestive of TB to first care seeking, receiving of TB diagnosis and subsequent initiation of TB treatment; types of providers (allopaths/non-allopaths/chemist/informal) and sectors (public/private) used in the entire pathway.

### Data management

Patients’ personal details were not entered in the data sheet but compiled separately by generating ‘unique patient ID codes’. These ID codes were used for identification of patient information during storage of each audio files (along with other patient-specific data) and during analysis, thus maintaining anonymity of data. All interview audio files, notes, consent forms and final lists containing unique ID codes with corresponding patient identification information e.g. name, address etc. were safely stored in a repository with restricted access. Password protected electronic back up of the same was done creating a database on the internet, accessible only to senior researchers from the team.

Three levels of data checking were undertaken for quality assurance. The first level check was an inter-researcher exchange of interview audio files, notes and quantitative data sheets with other pairs. Any discrepancy in responses was noted and discussed between the researchers for consensus or rectification. At the second level—25% of the first 10 interviews undertaken by each pair of researchers were rechecked by two senior researchers of the team (EL, AP). A final level—review of 10% interviews was conducted by a consultant to the study (SR).

Quantitative and qualitative data were entered in CSPro v5 and analyzed using Statistical Package for the Social Sciences (SPSS) v19. All the data entered into CSPro were crosschecked for data entry errors independently by two researchers (EL, AP).

### Data Analysis

**The following operational definitions were used for analysis of data: New TB patients:** Patients who had never been diagnosed with TB and/or treated for TB.

**Retreatment TB patients:** Patients who had previously been treated for TB, and were diagnosed with recurrent TB during or six months prior to the study.

Total pathway of the TB patient was the sum total of days between:

1. onset of symptoms suggestive of TB and first contact with any health care provider to,
2. first contact with any health care provider and receiving PTB diagnosis to,
3. diagnosis of PTB and the initiation of anti-TB treatment.

**First care seeking delay:** Fifteen days or more from the onset of symptoms suggestive of TB to first contact with any health care provider, as per the Standards for TB Care in India (STCI) guidelines [25].

**Diagnostic delay:** Fifteen days or more from the first contact with any health care provider to receiving PTB diagnosis, as per the RNTCP guidelines [26].

**Treatment initiation delay:** Seven days or more between the diagnosis of PTB to the initiation of anti-TB treatment, also as per the RNTCP guidelines [26].

**Total pathway delay:** A conservative estimate of greater than 35 days was used to define a cut-off value from the time interval between the onset of symptoms suggestive of TB to initiation of anti-TB treatment.

Pathway durations and delays are represented as means, medians and ranges of observed days.

Patients who demonstrated extreme delays based on the median value of the pathway segment depicting delay (in days) were defined as outliers. Based on the median, outliers were considered as 24 days or more for first care seeking delay, 46 days or more for diagnostic delay and 12 days or more for treatment initiation delay.

Patients with a) no delay vs. delay, b) all three delays vs. outliers (median) were compared using univariate analysis. Continuous variables viz. age, number of providers seen and pathways were analysed using independent T-test. Categorical variables including demographics (gender, education, occupation, literacy level), symptom/s (cough with fever with/without other symptom/s, cough without fever with/without other symptom/s, non-cough symptom/s with/without fever), classification as new/retreatment patients and public/private sector details were analysed using chi-square test. *P* values ≤ 0.05 have been considered to be statistically significant.

Italicised patient narratives are verbatim translations of open-ended responses. These are used to reflect reasons for delays and provide a qualitative fabric to the quantitative data presented.

### Ethics consideration

The study was reviewed and approved by the Institutional Ethics Committee (IEC) of the Foundation for Medical Research vide IEC no. FMR/IEC/TB/01/2013. The consent forms used in the study were approved by the IEC. For the initial HH survey a verbal consent was obtained from patients, while for the in-depth interviews a written informed consent was obtained, including consent for digital audio-recording and note-keeping, prior to participation in the study. Consent for interview of minors was taken from their care-givers (parents/guardians).

## Results

### Patient demographics and characteristics

Of the 76 patients interviewed as seen in Table 1, more than half the patients (54%, n = 41) were in the young age group of 16–34 years and the proportion of males was higher than females (61%, n = 46). Forty-one percent of patients (n = 31) were unemployed at the time of interview and less than half the patients (42%, n = 32) had up to secondary level education. Among the 54 patients who reported their family income, 80% (n = 43) were living below the poverty line (estimated using the power purchasing parity). Fifty-seven percent patients (n = 43) did not admit to having a past history of TB. Thirty-two percent patients (n = 24) reported any form of addiction. Seventeen percent TB patients (n = 13) reported co-morbidities, among these six were diabetics and four were people living with HIV (PLHIV)

**Table 1:** Demographics and characteristics of the patients (N = 76).

**Age (years) n (%)**	
≤ 15	5 (6)
16–34	41 (54)
35–54	15 (20)
≥ 55	15 (20)
**Gender n (%)**	
Male	46(61)
Female	30(39)
**Occupation n (%)**	
Unemployed	31 (41)
Salaried	11 (14)
Self employed	10 (13)
Daily wage/casual	6 (8)
Housewife	9 (12)
Students	9 (12)
**Education n (%)**	
Illiterate	14 (18)
Primary (< 4th standard)	8 (11)
Secondary (< 9th standard)	32 (42)
Senior secondary (SSC/HSC)	16 (21)
Graduate and above	4 (5)
Not available	2 (3)
**Current monthly household income**^**±**^ **n (%)**	
Below poverty line	43 (80)
Above poverty line	11 (20)
**Classification of patients n %**	
New (no history of TB)	43 (57%)
Retreatment (past history of TB)	33 (43%)
**Use of addictive substances n %**	
Yes	24 (32)
No	52 (68)
Type of addictive substances consumed^*^ **n**	
Cigarette/beedi	8
Khaini/tobacco	14
Alcohol	12
Recreational drugs	1
**Co-morbidity n %**	
Yes	13 (17)
No	63 (83)
**Type of co-morbidity**^#^ **n**	
HIV/AIDS	4
Diabetes	6
Hypertension	1
Silicosis	1
Others	2

*some patients had more than one addiction

# some patients had more than one co-morbidity; ± only 54 patients reported family income/month.

### Total Pathway for TB care: Duration and Delay

The mean duration for the total pathway to TB care was 65 days, with a difference of eight days between new (60 days) and retreatment TB patients (68 days).

The proportion of new patients among outliers was similar (55%, n = 12/22) as compared to retreatment patients (45%, n = 10/22). Three out of the five (60%) children in the study had total pathway delay beyond 35 days (data not presented).

In addition, from the 29% outliers (n = 22/76) in the total sample, four patients had experienced all three delays.

No associations were noted between age, gender, education, occupation and type of first provider seen among outliers and non-outliers.

### First care seeking: Duration and Delay

**Fig 2:**
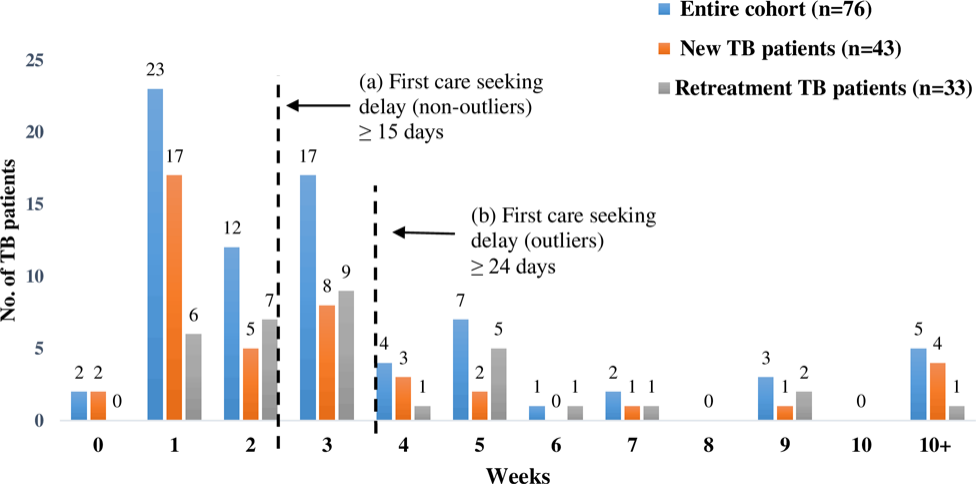
First Care Seeking: Duration and Delays. The figure represents the time taken from onset of symptoms suggestive of TB to first contact with any health care provider. The serrated lines depict the durations that reflect (a) delay and (b) outliers.

The mean duration of first care seeking for new (24 days) and retreatment patients (25 days) was similar (Table 2). Higher proportion of new TB patients (60%, n = 34/57) than retreatment patients (40%, n = 23/57) were seen among non-outliers. While comparable proportions of new (47%, n = 9/19) and retreatment patients (53%, n = 10/19) were seen in the outliers (Fig 2). Similar retreatment patients (50%, n = 10/20), though showing delay, were non-outliers than new patients (50%, n = 10/20). (p = 0.869).

**Table 2:** Duration for first care seeking.

	Entire cohort	New	Retreatment
< 15 days	37	24 (65%)	13 (35%)
≥ 15 days	39	19 (49%)	20 (51%)
Mean in days (range)	24 (0–191)	24 (0–191)	25 (0–169)
Median in days	15	10	15

No significant associations were noted between age, gender and education and both non-outliers and outliers for first care seeking delay, though the outliers group comprised of patients from the age group of 16–34 years, secondary level education, unemployed and those seeking care from the private sector. Fifty-eight percent patients (n = 25/43) below the poverty line experienced first care seeking delay, and 52% of these (n = 13/25) were outliers. There was also no significant association between the type of initial symptoms reported by the patients and first care seeking delay (data not presented).

The key factor responsible for delay in first care seeking after onset of symptom/s was the patient’s perception of his/her symptom/s; this was seen among both new and retreatment groups. Twenty-five patients delayed first care seeking citing reasons such as “*symptoms were not serious*” and that the symptoms “*may get relieved on their own*” (Table 3). Financial constraints and lack of time reported as major reasons by very few patients. Fifty percent patients (n = 12/24) who reported addictions such as smoking, alcohol etc. had first care seeking delay (data not presented).

**Table 3:** Reasons for delay^†^.

	Patients showing delay	
**First care seeking delay**	**Non-outliers 15–23 days (n = 20)**	**Outliers ≥24 days (n = 19)**
thought symptoms were not serious and would relieve on their own	16	9
attributed symptoms to other causes (weather, pollution, common cough and cold)	8	5
tried self-medication and home remedies	8	1
did not want to miss work	3	3
due to nature of work	3	3
financial constraints	0	1
lack of time	0	1
**Diagnostic delay**	**Non-outliers 15–45 days (n = 21)**	**Outliers ≥46 days (n = 20)**
**Patient-related factors**		
provider shopping	1	7
delay in approaching provider after leaving a previous provider	13	7
migration	2	5
financial constraints	2	4
delay in getting tests done and collecting results	0	1
refusal to get tests done	0	2
use of over-the-counter drugs (self-medication)	1	0
**Provider-related factors**		
advising symptomatic treatment for long duration without advising non-TB relevant tests	16	7
delay in advising TB-relevant tests	14	11
wrong diagnosis	2	2
referral	5	3
11 patients delayed due to patient and provider related factors, 13 due to provider related factors and 5 due to only patient related factors.		
**Treatment initiation delay**	**Non-outliers 8–11 days (n = 7)**	**Outliers ≥12 days (n = 6)**
**Patient-related factors**		
provider shopping	5	1
denial of diagnosis	1	0
migration	1	1
other complications	0	1
**Provider-related factors**		
referral by diagnosing provider to another provider for initiation of treatment	6	3
poor provider behaviour	1	0
long symptomatic treatment as patient was sputum negative (despite TB diagnosis by previous provider)	0	1
3 patients delayed due to patient related factors and 5 due to both-patient and provider related factors		

† some patients had no response or more than one response.

An example of perception of symptoms and self-medication leading to first care seeking delay is exemplified in the following excerpt:

*“He had cough along with fever for one month…We thought that this was* ***probably because he almost always has a cough***, *hence he has a cough and fever this time…So we bought Crocin and cough syrup from the medical [sic]*, *but since he did not feel any better even after 2 days*, *we took him to our family doctor*.”(Mother, 7-year old male patient)

### Diagnosis: Duration and Delay

The duration for TB diagnosis contributed most to the total pathway for TB care.

**Fig 3:**
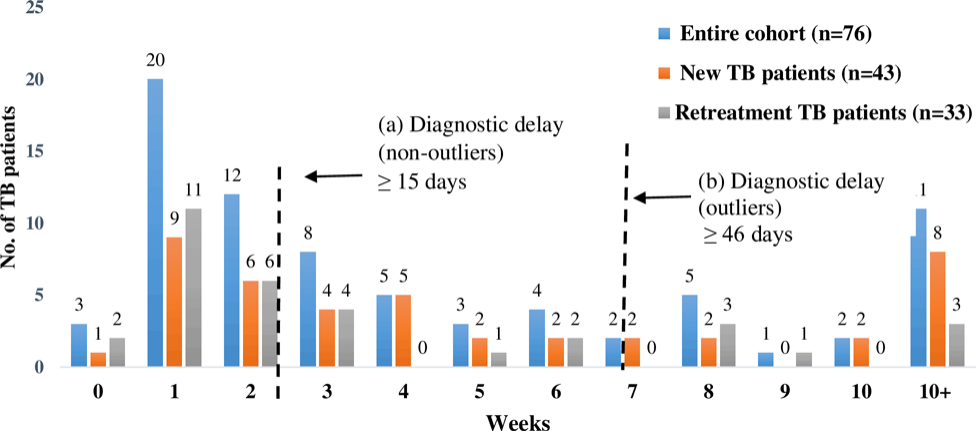
Diagnosis: Duration and Delays. The figure represents the time taken from first contact with any health care provider to receiving PTB diagnosis. The serrated lines depict the durations that reflect (a) delay and (b) outliers.

Two-thirds of those with diagnostic delay (63%, n = 27/43) were new patients in comparison to 42% (n = 14/33) retreatment patients. (Fig 3); and the average diagnostic duration was also much more for new patients (42 days) compared to retreatment patients (28 days) (Table 4). Thirteen out of the 20 outliers were new TB patients. The characteristics of the outliers for diagnostic delay were similar to those for first care seeking delay. Also five of the seven patients who reported delay in diagnosis due to relocation were outliers for diagnostic delay.

**Table 4:** Duration for diagnosis.

	Entire cohort	New	Retreatment
< 15 days	35	16 (46%)	19 (54%)
≥ 15 days	41	27 (66%)	14 (34%)
Mean in days	36 (range: 0–227)	42 (range: 0–169)	28 (range: 0–227)
Median in days	19	25	12

Sixty-seven percent of female patients (n = 20/30) reported diagnostic delay in comparison to 46% males (n = 21/46) (p = 0.05) (data not presented in tables). Age, education and occupation were not associated with either non-outliers or outliers. Among the 54 patients who reported their family income, half the patients below poverty line (51%, n = 22/43) had diagnostic delay, 60% (n = 13/22) of whom were outliers, while 36% patients (n = 4/11) above the poverty line experienced diagnostic delay with only one outlier (data not presented in tables).

Diagnostic delay was not associated with the type of first provider (public/ private) approached (data not presented in tables).

Both patients and providers contributed to the diagnostic delay among non-outliers and outliers (Table 3). Delay on the part of patients in seeking further care seeking after leaving the previous provider and delay on the part of providers in advising relevant tests for diagnosis of TB and giving symptomatic treatment to patients for long periods featured as prominent reasons for diagnostic delay.

### Treatment initiation: Duration and Delay

**Fig 4:**
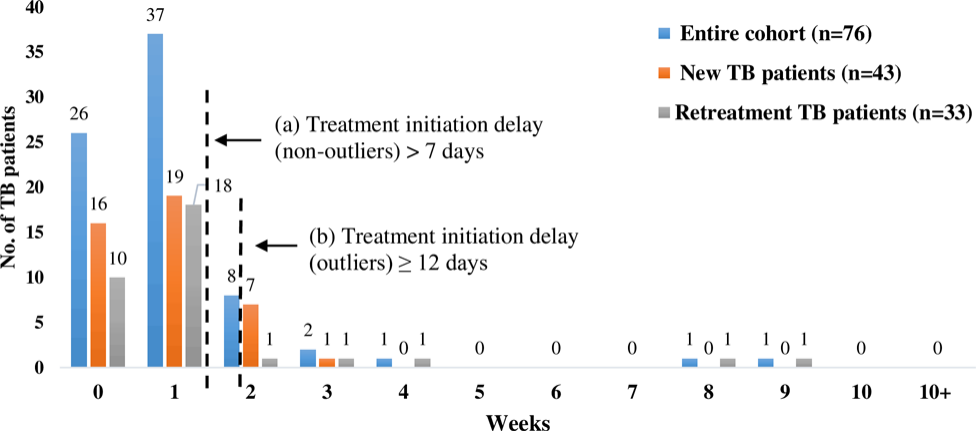
Initiation of Treatment: Duration and Delays. The figure represents the time taken from diagnosis of PTB to the initiation of anti-TB treatment. The serrated lines depict the durations that reflect (a) delay and (b) outliers.

Treatment initiation was noted to be the least among the three durations. The mean treatment initiation duration for retreatment patients (7 days) was more than twice that of new patients (3 days), though the percentage of new patients was higher (62%) among those with delays (Table 5). Four out of six outliers were retreatment patients. (Fig 4)

**Table 5:** Duration of treatment initiation.

	Entire cohort	New	Retreatment
< 7 days	63	35 (56%)	28 (44%)
> 7 days	13	8 (62%)	5 (38%)
Mean in days	5 (range: 0–58)	3 (range: 0–17)	7 (range: 0–58)
Median in days	1	1	2

Age, gender, education, occupation and initial symptoms had no association with the duration of treatment initiation and outliers. However more retreatment patients had treatment initiation delay compared to new patients. (p>0.05). The characteristics of the outliers for diagnostic delay were similar to those for first care seeking and diagnostic delay. Twenty-one percent patients (n = 9/43) living below the poverty line reported treatment initiation delay including two unemployed patients, as compared to 18% patients (n = 2/11) above the poverty line. Provider-related factors were responsible for treatment initiation delay in six of the 7 non-outliers. Active referrals by the diagnosing provider to another provider for treatment initiation constituted the major reason for delay; among patient-related factors, provider shopping was the most common. (Table 3).

Below is a quote from a patient, highlighting provider shopping and denial of diagnosis:

*“When I approached Dr*. *X*, *he gave me medicines but I did not feel better after taking them*. *So I went back and told him*, *that’s when he advised a Chest X-ray*. *On receiving the report***, *he told me I have TB*, *but I did not believe him*.** *And went to Dr*. *Y*, *who diagnosed me with Typhoid and started with a treatment of 10 days…But I still did not feel any better*, *and my fever had increased…Finally I approached Dr*. *Z*, *who saw my earlier test reports and diagnosed TB*. “(27-year old male patient)

### Provider switching

**Fig 5:**
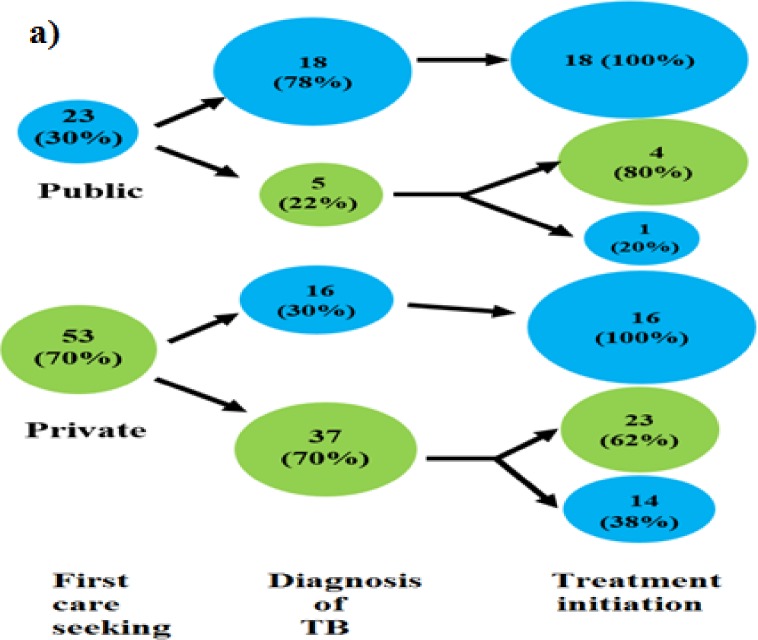
Provider switching at different stages of the TB patient care pathway: a) between public and private sector; b) between allopaths and non-allopaths^†^. The figures depict the patient behaviour in seeking TB care at different stages of the pathway and preference for accessing the public or the private sector, and allopaths or non-allopaths.

The first point of care for a large proportion of patients (70%, n = 53/76) was the private sector (Fig 5A); this included 45% patients (n = 24) approaching non-allopaths, followed by 25% (n = 13) allopaths and 15% (n = 8) chemists. The qualifications of the first private provider consulted by 15% (n = 8/53) of patients were not available.

There was a striking shift of patients from the private sector to the public sector and likewise from non-allopaths to allopaths from the onset of symptom/s to initiation of treatment (Fig 5A & 5B). Forty-five percent of patients (n = 34/76) were diagnosed in the public sector and of those diagnosed in the private sector, a majority (64%, n = 27/42) were by allopaths.

However for treatment initiation, a shift of almost 40% patients to the public sector and a prominent shift of 80% from non-allopaths to allopaths was observed. (Fig 5A & 5B) Sixty-six percent patients (n = 50/76) had been initiated on treatment in the public sector. Overall in the private sector, 77% (20/26) patients were initiated on treatment by allopaths.

A majority of the patients with treatment initiation delay (85%, n = 11/13) had been diagnosed with TB in the private sector (p = 0.02), and shifted or were shifted to the public sector for treatment initiation. (69%, n = 9/13).This delay in treatment initiation can therefore be attributed to referral-related movement.

### Analysis on the basis of recall period

The time between onset of symptoms to interview was compared between groups of patients who had recall periods of less than and over 6 months (180 days). Sixty-six percent (n = 50/76) of the patients were interviewed within 6 months of onset of symptoms and 34% (n = 26/76) beyond 6 months. Patients with a delay of more than 6 months between symptoms and interview (79 days vs. 37days) showed significantly longer pathways (p≤0.05) compared to patients with recall period of 6 months or less. The patients were further analysed based on their past history of TB and recall period. Amongst new patients, first care seeking (33 days vs. 10 days), diagnostic (53 days vs. 25 days), and total pathways (89 days vs. 37 days) were significantly higher for patients with longer recall periods (p≤0.05); whereas for retreatment patients, the treatment initiation (9 days vs. 1 day) and total pathway (67 days vs. 37 days) were significantly higher for the same group. (p≤0.05)

## Discussion

The findings from our study on pathways to care for TB in vulnerable urban settings in Mumbai, generally complement the findings of several studies undertaken in India [27, 28, 29] and elsewhere [30, 31, 32, 33]. The largest contributor to the total duration of 65 days extending from the beginning of symptoms to the initiation of treatment, as reported by patients, was the time spent on obtaining the diagnosis of TB.

The first care seeking duration of 24 days was, however seen to be considerably shorter than those reported from studies undertaken in Low and Middle-Income Countries (LMICs). [14] It is possible that patients’ symptoms may have begun substantially earlier but were perceived as distressing much later. Additionally, chronic renal insufficiency [34], HIV positivity and infection with tuberculosis lineage 7 ancient strains [35] have all been associated recently in prolonged patient delay in seeking treatment. Lastly, in a city where high levels of pollution and allergens are a constant feature, the occurrence of cough with or without fever is considered a non–serious symptom and not worthy of out-of-home provided care.

Both patient- and provider-related factors were responsible for the delay in diagnosis of TB. Our findings confirm the provider-related factors documented by earlier studies, [15, 36] which include long symptomatic treatment without ordering relevant tests for TB, misdiagnosis, poor provider behaviours and delays due to referral-related movement to the public sector. While diagnostic duration in retreatment cases was 28 days as compared to 42 days for new cases, both were well beyond the recommended period of 15 days for an effective TB control programme.

An important finding of our study was the similarity in duration of first care seeking among new (24 days) and retreatment (25 days) patients; this has been reported once before for Malaria. [37] The absence of an amnestic response in retreatment patients could be a result of denial of recurrence of the disease, or the misguided belief that TB can strike only once in a lifetime. There is a strong biological basis for recurrence / relapse of TB particularly in the early years after the first episode [38] which should be taken into account when providing counseling to a TB patient at the time of completion of treatment.

This study recorded the least contribution to the care pathway from treatment initiation duration. The duration ranged from 0–58 days with only 17% (n = 13/76) patients experiencing a delay beyond 7 days. The contrast between this and recent studies undertaken in West Bengal and Andhra Pradesh [39] where treatment initiation delay was longer, may be attributed to the perception of a better performing public health system in Mumbai. This explanation is further strengthened by the findings of this and other studies of the shift of patients from the private to the public sector in the course of the care pathway [40, 41, 42] our study showed that 64% of patients ended up with the public sector for treatment, despite 70% seeking help initially in the private sector. This shows the important role played by the RNTCP particularly for providing TB treatment to the poor and vulnerable in a context like Mumbai, which has a huge private sector presence. While there is a need to greatly augment the capacity and quality of the private sector, there is an equal need to continue strengthening the public sector's capacity to provide stewardship and help control the disease. The findings also point to the need for an immediate engagement of certified non-allopaths and chemists in TB control activities, since they are accessed for first care seeking in vulnerable locations [43]. The importance of the know-do gap in these providers highlighted by the seminal pilot study by Das *et al* [44] needs to be ascertained and the gap redressed through a combination of both regulation and education. [45]

While delay was not seen to be associated with age, education, occupation or type of health facility first visited, a significant association was noted between the female gender and delay in diagnosis; these are similar to findings of prior studies from Vietnam, Nepal and Ghana. [46, 47, 48] Whether this was due to a lower index of suspicion of TB among providers for women or behavioral patterns of women dictated by their inherent care-giving roles remains speculative. [49]

Eighty percent of patients, whose socio economic status could be determined, were living below poverty line and 40% were unemployed at the time the study was undertaken. Their vulnerability was further emphasized by the findings of 29% patients being outliers, four of whom experienced all three delays. An amalgamation of lack of education, finances and time can push such vulnerable groups towards delays and finally even poor outcomes. An insightful viewpoint is provided by Andrews *et al* [50] through a review of data on TB disparities in India and the wealth distribution of known TB factors. It concludes that a greater impact could be achieved if efforts are targeted at those living below the poverty line than a population wide strategy with a weave of equity considerations in TB control plans.

Though diagnostic delays can be reduced somewhat through evidence-based education of generalist providers and provision of accessible and affordable point-of care technologies, the help seeking delay hinges heavily on patient behavioural patterns, their perception and awareness of consequences of TB infection [51] and their environmental ecology. Prioritizing the value of well-being, and bringing about visible improvement in environmental health (sanitation, waste disposal, pollution control, housing) should shake the lethargy imposed by a sustained state of feeling unwell (as opposed to ill).

This view should be directed to seek a balance between interventions directed at provider engagement [52] and those targeted to raise community awareness of disease and foster positive behavioural change. [53]

In the light of the study designed it is appropriate to mention the recall bias due to differential recollection of the patients of their entire pathway. Since the design of this study was retrospective with a recall period of six months, it could be expected that patients would have underestimated the time they took to access care. However analysis of the patients based on the time from the onset of symptoms to interview showed that patients with a longer recall period possibly overestimated their pathway durations, as compared to patients with shorter recall.

Other limitations included not being able to interview all the patients identified from the HH survey. Patients unavailable due to reasons such as hospitalization or unavailable at the time of researchers’ visit were contacted more than once for the interview. A maximum of three attempts were made to contact and interview such patients. These patients may have had longer pathways to care compared to the patients included in the final sample.
