# Correction: Durations and Delays in Care Seeking, Diagnosis and Treatment Initiation in Uncomplicated Pulmonary Tuberculosis Patients in Mumbai, India

**DOI:** 10.1371/journal.pone.0160796

**Published:** 2016-08-03

**Authors:** Nerges Mistry, Sheela Rangan, Yatin Dholakia, Eunice Lobo, Shimoni Shah, Akshaya Patil

In Fig 5, Fig 5B is missing. Please see the corrected Fig 5 here.

**Fig 5:**
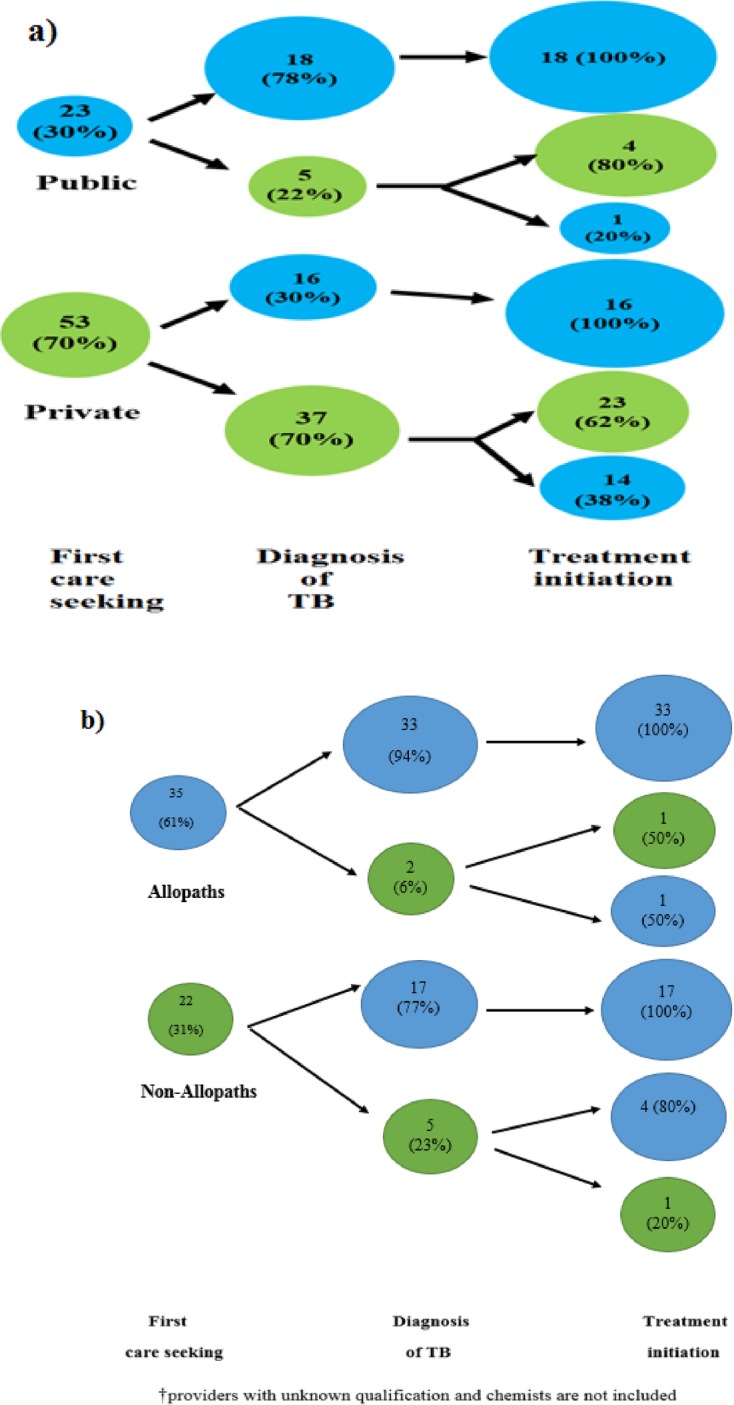
Provider switching at different stages of the TB patient care pathway: a) between publicand private sector; b) between allopaths and non-allopaths†. The figures depict the patient behaviour in seeking TB care at different stages of the pathway and preference for accessing the public or the private sector, and allopaths or non-allopaths.
